# Kelch Domain of Gigaxonin Interacts with Intermediate Filament Proteins Affected in Giant Axonal Neuropathy

**DOI:** 10.1371/journal.pone.0140157

**Published:** 2015-10-13

**Authors:** Bethany L. Johnson-Kerner, Alejandro Garcia Diaz, Sean Ekins, Hynek Wichterle

**Affiliations:** 1 Project A.L.S./Jenifer Estess Laboratory for Stem Cell Research, New York, New York, United States of America; 2 Center for Motor Neuron Biology and Disease, Columbia University Medical Center, New York, New York, United States of America; 3 Departments of Pathology and Cell Biology, Neurology, and Neuroscience, Columbia University Medical Center, New York, New York, United States of America; 4 Columbia Stem Cell Initiative, Columbia University Medical Center, New York, New York, United States of America; 5 Collaborations in Chemistry, Fuquay-Varina, North Carolina, United States of America; University of Vienna, Max F. Perutz Laboratories, AUSTRIA

## Abstract

Patients with giant axonal neuropathy (GAN) show progressive loss of motor and sensory function starting in childhood and typically live for less than 30 years. GAN is caused by autosomal recessive mutations leading to low levels of gigaxonin (GIG), a ubiquitously-expressed BTB/Kelch cytoplasmic protein believed to be an E3 ligase substrate adaptor. GAN pathology is characterized by aggregates of intermediate filaments (IFs) in multiple tissues. To delineate the molecular pathway between GIG deficiency and IF pathology, we undertook a proteomic screen to identify the normal binding partners of GIG. Prominent among them were several classes of IFs, including the neurofilament subunits whose accumulation leads to the axonal swellings for which GAN is named. We showed these interactions were dependent on the Kelch domain of GIG. Furthermore, we identified the E3 ligase MYCBP2 and the heat shock proteins HSP90AA1/AB1 as interactors with the BTB domain that may result in the ubiquitination and subsequent degradation of intermediate filaments. Our open-ended proteomic screen provides support to GIG’s role as an adaptor protein, linking IF proteins through its Kelch domain to the ubiquitin pathway proteins via its BTB domain, and points to future approaches for reversing the phenotype in human patients.

## Introduction

Giant axonal neuropathy (GAN) is a rare pediatric neurodegenerative disease, first described in 1972 by Asbury and Berg [1, 2]. It is best known for the “giant” axons caused by accumulations of intermediate filaments. The disease is a progressive sensorimotor neuropathy affecting both the peripheral (PNS) and central nervous systems (CNS), with onset around age 3 years and death by the third decade [3]. GAN results from recessive mutations in the *GAN* gene encoding gigaxonin (GIG) [4]. Precisely how these mutations cause the disease remains to be determined, in part because the function(s) of GIG remain unclear [5].

GIG is a member of the BTB (Bric-a-brac, Tramtrack and Broad)/Kelch superfamily [4]. Members of this family have a shared domain organization and show ~25% sequence identity [6] but their biochemical functions remain uncertain. The best-studied BTB/Kelch protein, Keap1, serves as a substrate adaptor protein to target the Nrf2 transcription factor for degradation by the 26S proteasome [7]. This occurs when the Kelch domain interacts with Nrf2 while the BTB domain binds the E3 ligase cullin 3 (Cul3) leading to Nrf2 ubiquitination. In overexpression studies, GIG has been shown to associate with Cul3 and ring box protein 1 (Rbx1) to form a functional ubiquitin ligase complex [7]. Moreover, GIG was one of the proteins that bound a Cul3 bait during proteomic analysis of the cullin-RING ubiquitin ligase network [8].

Disorganization of the neurofilament network is a feature of several neurodegenerative disorders, including amyotrophic lateral sclerosis (ALS), Parkinson’s disease and axonal Charcot-Marie-Tooth disease [9, 10]. In GAN such changes are often striking: peripheral nerve biopsies show enlarged axons with accumulations of neurofilaments referred to as “giant axons” [1]. Interestingly, IFs also accumulate in other cell types in patients. These include desmin in muscle fibers, glial fibrillary acidic protein (GFAP) in astrocytes, and vimentin (VIM) in multiple cell types including primary cultures of biopsied fibroblasts [11–13]. Given the homology of GIG to Keap1 and its reported association with Cul3, a hypothesis that GIG acts as an E3 ligase substrate adaptor for targeting IFs to the proteasome has been proposed [7]. A yeast two-hybrid screen identified several microtubule-associated proteins as binding partners of GIG but no interactions with IFs were reported [14]. More recently, it was observed that GIG interacts with VIM, neurofilament light chain (NEFL) and peripherin (PRPH) and that proteasome inhibition reversed the clearance of IF proteins in GIG over expressing cells [15], providing a first possible link between GIG and IFs. We were interested in determining if other IF proteins are part of this complex and most importantly if the interaction with IFs occurs through the Kelch domain of GIG, which would be necessary for a ubiquitination complex to form. We demonstrate that GIG binds to several classes of IFs, including the neurofilament subunits whose accumulation leads to the axonal swellings for which GAN is named. Additionally, we demonstrate that these interactions are dependent on the Kelch domain of GIG. Finally, we identified a set of GIG binding partners that may interact with BTB domain and target IFs and other substrates for degradation.

## Results

**Fig 1:**
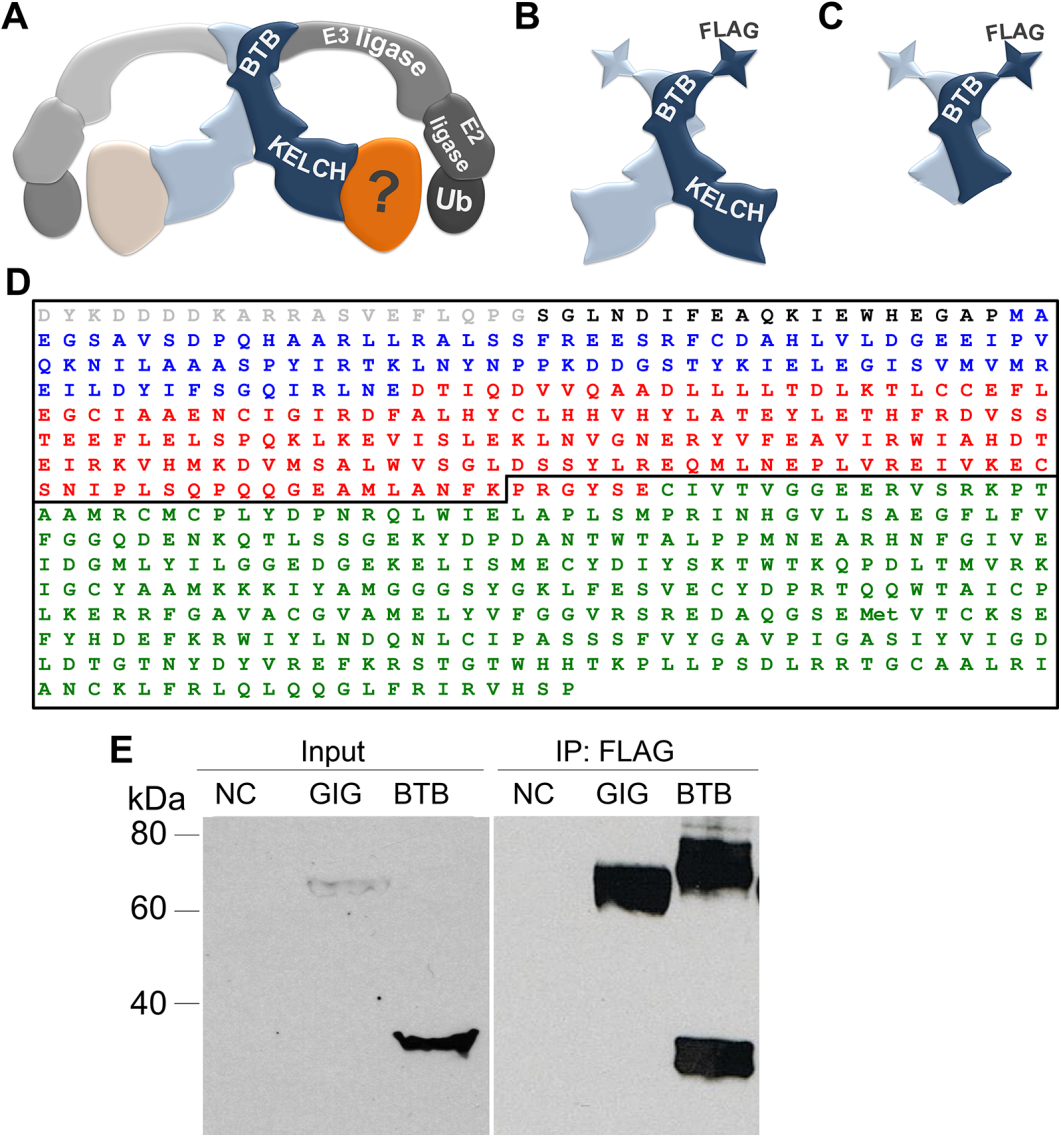
Design of proteomic bait to determine the binding partners of GIG. (A) GIG is a BTB/Kelch protein, and these domains are separated by an intervening BACK domain. GIG is thought to dimerize through its BTB domain [16]. The BTB domain has also been shown to bind to E3 ligases, potentially for ubiquitin tagging of Kelch targets. To identify potential substrates for degradation/modification (Kelch binding partners) we expressed flag-tagged full-length GIG (B) or its BTB domain (C). (D) Sequence of proteomic baits with key regions identified by color. The constructs expressed included a single flag tag and linker (grey), Biotag (black), BTB domain (blue), BACK domain (red), and Kelch domains (green). The location of the truncation for the BTB bait shown in (C) is indicated by the black line at amino acid 268, towards the N-terminus of the BACK domain. (E) Flag-tagged constructs were expressed in HEK293 cells and affinity purified with an anti-Flag antibody (abbreviated as F). The membrane was then probed with anti-Flag tag antibodies. Untransfected cells (NC) were included to control for non-specific binding to the antibody or beads. Flag-tagged GIG was ~62 kDa, while the BTB domain was ~34 kDa and forms a dimer in the immunoprecipitation sample that was ~70 kDa.

Few binding partners of GIG have been previously reported, and no large-scale proteomic analysis has been performed to determine its cellular interactions. To understand the normal function of GIG and the consequences of its loss of function in GAN, we sought to better understand the protein complexes that GIG forms. By homology to Keap1, we reasoned that GIG would interact with its substrates through the Kelch domain and link to the ubiquitin ligases through its BTB domain (Fig 1A). We therefore constructed a full length and a truncated version of the GIG protein lacking its Kelch domain and performed an open-ended proteomic analysis using affinity purification of the control and truncated GIG proteins expressed in human embryonic kidney (HEK293) cells followed by mass spectrometry (MS) (Fig 1B and 1C). The constructs included a C-terminal FLAG-tag, linker and biotag, and the Kelch domain was removed by truncating the protein at the N-terminus of the BACK (BTB and carboxy-terminal kelch) domain (Fig 1D). This truncated product contains the full BTB domain as well as a portion of the BACK domain; for simplicity, it will be referred to as the “BTB” domain throughout the manuscript. To validate expression of these constructs we performed immunoprecipitation of overexpressed FLAG-tagged full-length GIG or truncated GIG (BTB domain, as well as untransfected HEK293 cells to control for non-specific protein binding to the affinity purification beads (Fig 1E). Western blot with antibodies against the FLAG-tag revealed a specific band corresponding to full-length GIG at ~64 kDa, and a strong truncated BTB domain at ~34 kDa. We also detected a band at ~70 kDa in the BTB lanes, which likely reflected BTB homodimer formation [16].

**Fig 2:**
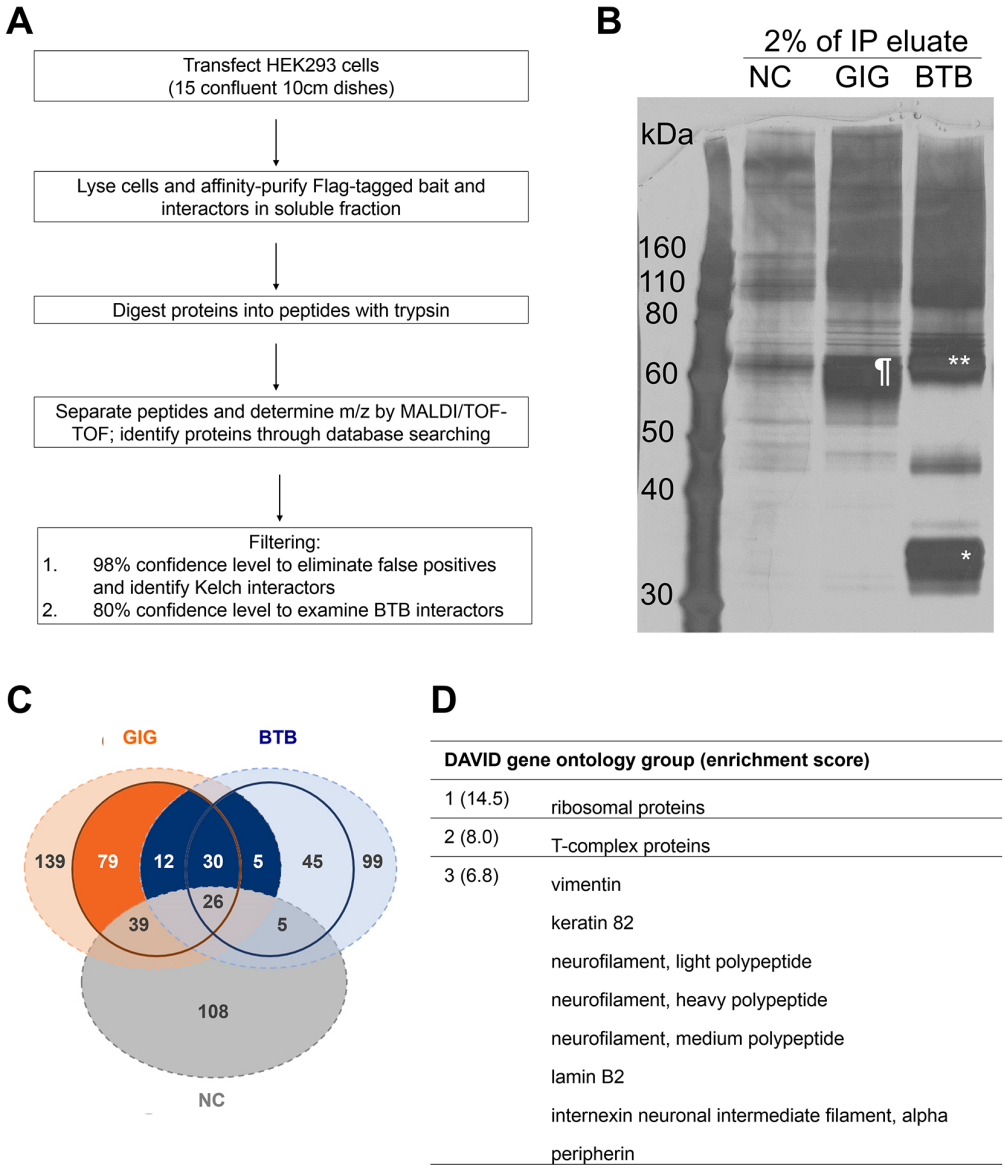
Proteomic screen to determine GIG’s binding partners. (A) Experimental and analytical work-flow. For each condition, 15 confluent 10 cm dishes of HEK293 cells were transfected. After 28 hours, cells were lysed and affinity purified using flag beads and the soluble fraction was isolated. Samples were sent to Applied Biomics for further processing and mass spectrometry analysis. Two percent of the immunoprecipitation eluate was run on silver stain (B) to confirm enrichment of the baits and successful pull-down of targets. Flag-tagged GIG (as indicated by ¶) was ~62 kDa, while the BTB domain was ~34 kDa (*, BTB dimers are indicated by **). Binding partners were analyzed by NanoLC MALDI-TOF/TOF and proteins were identified through database searching. (C) Venn diagram displaying data parsing categories, including GIG (orange), BTB (blue) and negative control (NC, grey) interacting proteins. Any proteins found in the NC were excluded from further analysis. The outer circle represents the 80% CI cut-off and the inner circle 98% CI cut-off. The dark orange area therefore represents the proteins that exclusively interacted with the Kelch domain at the 98% CI, and included 79 proteins. The dark blue area represents proteins that exclusively interacted with the BTB domain from both the 80 and 98% CI, and included 47 proteins total. (D) Gene ontology of hits from results in (C) as determined by DAVID cluster analysis. Top three clusters are shown along with enrichment scores in parentheses. See S2 Table for the full list of proteins from the DAVID analysis and complete list of ontology clusters.

We next utilized the constructs validated in Fig 1 to affinity purify proteins complexed with the control and truncated GIG and performed unbiased MS proteomic analysis. The procedure for the screen itself is outlined in Fig 2A. Following immunoprecipitation we examined enriched proteins by gel electrophoresis of 2% of the soluble eluate and detection of isolated proteins by silver stain (Fig 2B). As in Fig 1E, full-length GIG, BTB and BTB dimers were seen (at ~64, 34 and 60 kDa, respectively) as well as numerous pull-down products.

To determine the identity of the proteins complexed with full length and truncated GIG we performed MS analysis. MS was performed with Nano-LC fractionation, automated spotting, and MALDI-TOF/TOF (matrix-assisted laser desorption/ionization-time of flight). A complete list of hits is tabulated in S1 Table. For full-length GIG, at the 80% CI (confidence interval), 1553 peptides were assigned to 368 proteins. At the 98% CI, 1053 peptides were assigned to 189 proteins. For the BTB domain only, at the 80% CI (confidence interval), 1012 peptides were assigned to 252 proteins. At the 98% CI, 688 peptides were assigned to 115 proteins.

We took advantage of the high and low confidence hits to focus on a group of proteins that is most likely specifically interacting with the BTB and Kelch domains. As illustrated in the Venn diagram in Fig 2C, we excluded any hits that appeared at the 80% CI in the negative control (NC) condition (shown in grey). The orange areas represent proteins that interacted with full-length GIG; the outer circle symbolizes the 80% CI cut-off and the inner circle 98% CI cut-off. Given that we wanted to identify proteins interacting specifically with the Kelch domain, we further subtracted any proteins that interacted with the BTB domains at the 80% CI (blue areas). The dark orange area therefore represents the 79 proteins that exclusively and with high confidence (98% CI) interacted with the Kelch domain of GIG.

The blue areas represent proteins that interacted with the GIG fragment lacking the Kelch domain; the outer circle symbolizes the 80% CI cut-off and the inner circle 98% CI cut-off. Given that potentially meaningful BTB-binding partners would bind to both the full-length and truncated proteins, we assigned proteins as BTB-interacting (dark blue) if they were found in both full length and BTB-domain truncated pull-downs at the 80% CI and in either one of the pull downs at the 98% CI. The dark blue area represents the sum of these sets containing a total of 47 BTB-interacting proteins that we focused on for further analysis.

We first performed functional annotation to categorize the Kelch binding partners using the online gene ontology tool DAVID. The resulting analysis divided the hits into 4 gene groups, with enrichment scores ranging from 14.5 to 3.8 (Fig 2E; the complete list is provided in S2 Table) [17]. The most enriched gene group was ribosomal proteins (enrichment score 14.5). While ribosomal proteins have not been directly implicated in the pathogenesis of GAN, a previous study found that ribosomal proteins L29 and L37 (neither found in our study) were upregulated compared to control in a proteomic analysis of GAN patient fibroblasts [18]. The second highest-scoring group included multiple T-complex proteins, which have been proposed to associate with the ribosomal complex for nascent protein folding [19].

The third highest-scoring DAVID interaction group (enrichment score 6.8) included many intermediate filament proteins. Because of the GAN clinical phenotype we were particularly interested to see that full-length GIG bound several classes of IFs endogenously expressed in HEK293 cells [20] including VIM, keratin 82, lamin B2, NEFL, neurofilament medium chain (NEFM), neurofilaments heavy chain (NEFH), PRPH and α-internexin (INA). The fourth highest-scoring network (enrichment score 3.8) included ubiquitin A, B, and C as well as additional ribosomal proteins.

We then looked for functional interactions between hits using Ingenuity Pathway Analysis (IPA) software. The utility of this type of analysis is to determine possible interactions between hits that cluster based on their function, and the analysis considers both proteins found in our results as well as proteins in the IPA database. The hits from Fig 2C (79 Kelch-interacting proteins and 47 BTB-interacting proteins) were uploaded to the IPA program for generation of functional networks.

**Fig 3:**
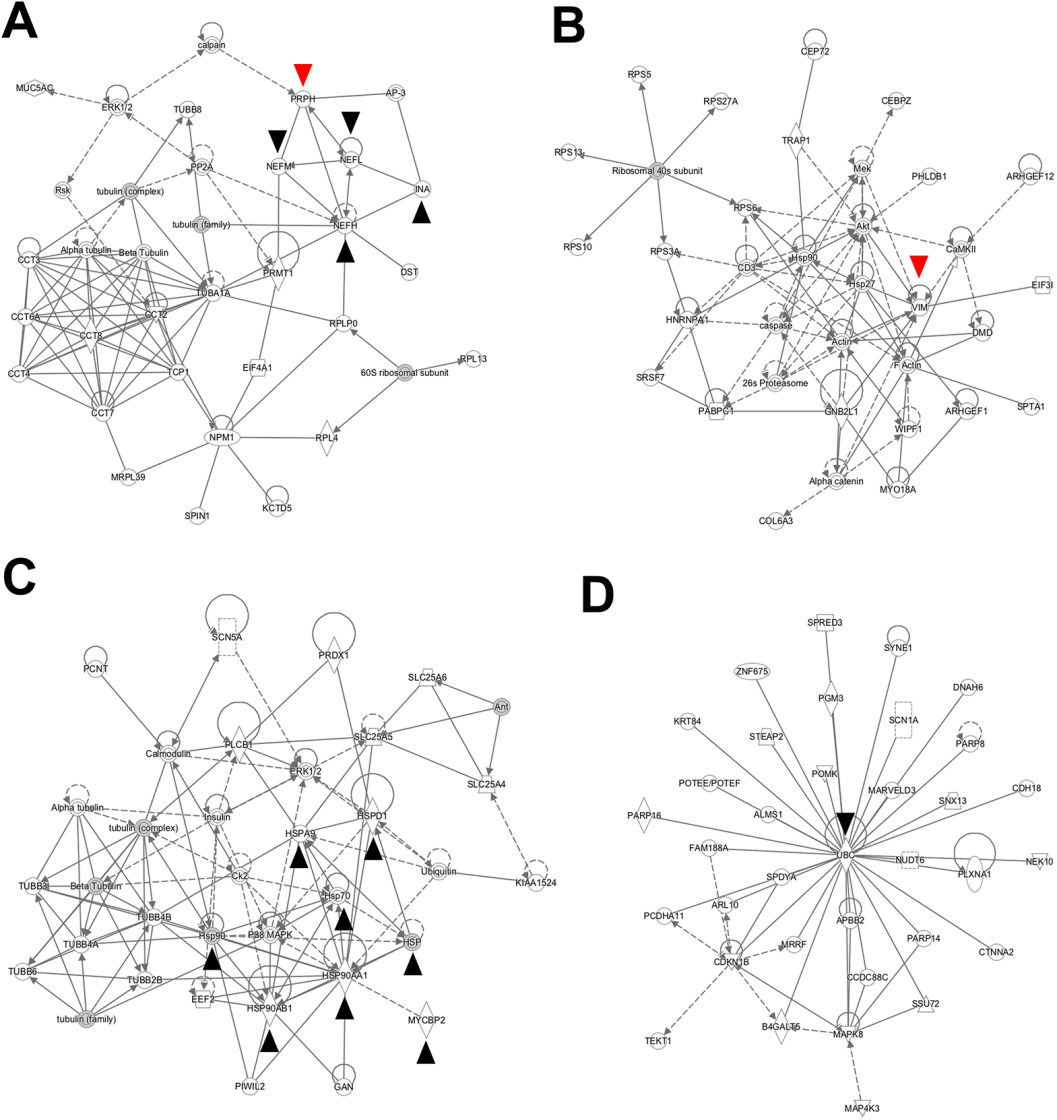
Ingenuity Pathways Analysis functional network for GIG-interacting proteins. (A) Top interaction network for Kelch hits includes PRPH, NEFL, NEFM and NEFH and α-internexin and multiple tubulin proteins. See main text for identification of proteins as experimentally found interactors from the MS versus validated interactors from the IPA database. The only relationships considered are those that are experimentally observed according to the IPA database. Solid lines indicate direct interactions; dotted lines indicate presumed indirect interactions. Looped arrows represent autoregulation. Black arrowheads indicate proteins that are mentioned in the manuscript, and red arrowheads indicate proteins with experimentally confirmed interactions (see Fig 4). S1 Fig describes additional details regarding the significance of shapes in the networks and their relationships to protein families. (B) Second-ranked interaction network, which includes VIM. (C) Top interaction network for BTB hits, noted to include multiple heat shock proteins and MYCBP2, as well as multiple tubulin proteins. (D) Second-ranked interaction network for BTB hits, which is centered on the ubiquitination protein UBC.

The resulting IPA networks for Kelch and BTB-interacting proteins are shown in Fig 3. Kelch-interactors were grouped into five networks. The top two networks are show in Fig 3A and 3B (the additional networks are shown in S2 Fig). The top network contained many intermediate filament proteins, including NEFL, NEFM, NEFH, PRPH and INA (Fig 3A). The second highest interaction group included VIM. This result suggests that GIG interacts with multiple intermediate filament proteins through the Kelch domain.

We next examined the 47 unique BTB binding partners to determine the significance of the interaction between GIG and intermediate filaments. BTB-interactors were grouped into four networks. The top two networks are show in Fig 3C and 3D (the other two networks are show in S3 Fig). Interestingly, the top interaction network of the BTB hits included multiple heat shock (HSPs) and tubulin proteins (Fig 3C). HSPs found experimentally included Hsp90 proteins, (HSP90AA1 and HSP90AB1). Based on the IPA database, proteins that have been shown to interact with experimental proteins include Hsp70, HSPA9, and HSPD1, as well as ubiquitin. Multiple tubulin proteins—but no tubulin-specific chaperone proteins—were found.

As the E3 ligase Cul3 has been proposed as a potential mediator of IF ubiquitination via GIG, we were interested to see that the E3 ubiquitin protein ligase MYC binding protein 2 (MYCBP2) was also an experimentally found interactor of the BTB domain in the top interaction network. The second highest interacting group (Fig 3D) was centered on ubiquitin C protein (UBC, not experimentally found but identified as a potential network hub via the IPA database). Therefore, the BTB domain of GIG may interact with ubiquitination machinery including MYCBP2 as well as HSPs.

**Fig 4:**
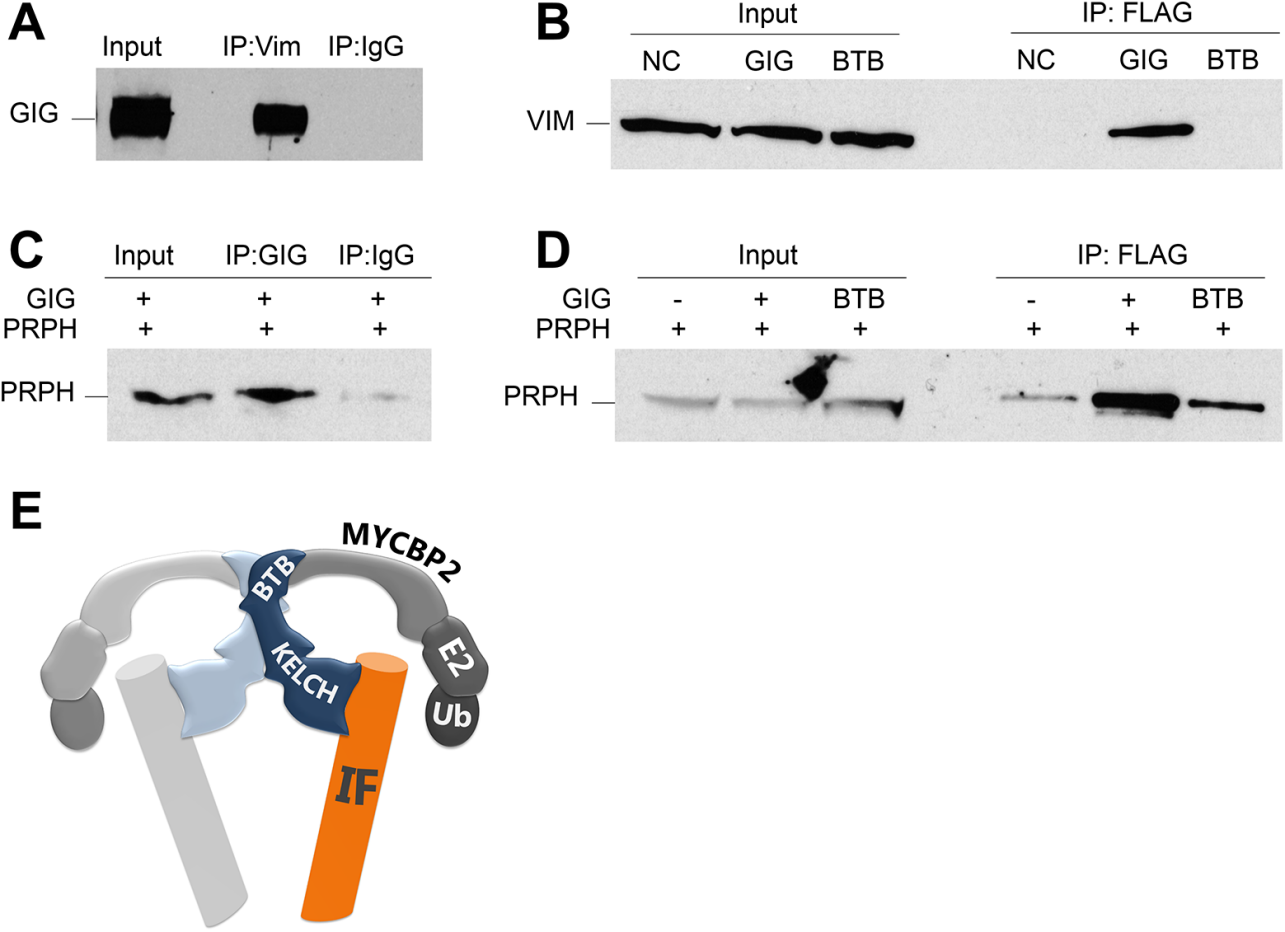
The Kelch domain of GIG interacts with intermediate filament proteins. (A) Endogenous VIM pulls down endogenous GIG. Indicated lysates were subjected to immunoprecipitation with either VIM antibody or control IgG (used as the mock condition throughout), and the membrane was probed sequentially for endogenous GIG and then VIM (not shown). (B) GIG pulls down endogenous VIM in a Kelch-dependent manner. Cell lysates transfected with the indicated constructs were subjected to immunoprecipitation with anti-Flag beads, and the membrane was probed sequentially for VIM and then GIG (not shown). (C, D) GIG pulls down overexpressed PRPH in a Kelch-dependent manner. Cell lysates were subjected to immunoprecipitation with anti-Flag beads or IgG, and the membrane was probed sequentially for PRPH then GIG (not shown). (E) Working model of GIG, a novel regulator of intermediate filaments.

Recently, Mahammad et al. (2013) reported interactions between FLAG-tagged GIG and NF-L, PRPH and VIM [15]. However, it has not been determined whether GIG interacts with these intermediate filament proteins via the substrate binding Kelch domain. To confirm the interaction between GIG and select intermediate filaments we performed immunoprecipitations of endogenous proteins. We first sought to confirm the interaction between endogenous GIG and VIM. VIM is abundantly expressed endogenously in HEK293 cells, and has been shown to be elevated in GAN patient fibroblasts [21]. Immunoprecipitation of endogenous VIM from HEK293 cells resulted in pull-down of endogenous GIG (Fig 4A). This interaction occurs through the Kelch domain of GIG since the BTB domain did not alone bring down VIM (Fig 4B). Subsequently, we confirmed the interaction between PRPH and GIG (Fig 4C), and showed that this interaction is similarly Kelch-dependent (Fig 4D), with levels of PRPH precipitated by the BTB domain closer to NC levels than that of full-length GIG. Unlike VIM, PRPH is expressed in sensory and motor neurons [22]. Our data provide strong evidence for an interaction between the Kelch domain of GIG and IFs whose levels and localization are strongly affected in GAN patients.

## Discussion

We performed an unbiased proteomic screen in cells expressing full-length and truncated versions of GIG to detect binding partners that might help explain the characteristic pathology of giant axonal neuropathy. We confirmed the ability of GIG to bind intermediate filament proteins deduced from overexpression studies and added several significant partners to the known list of interactors, while failing to validate others from the literature. We show that intermediate filaments interact with the Kelch domain of GIG, while its BTB domain binds to heat shock proteins and elements of the ubiquitin degradation pathway. Our data suggest that intermediate filament aggregation in patients with giant axonal neuropathy may result from the failure of mutant cells to manage proteostasis in the absence of a critical organizer of the required protein complexes.

The pathway results suggest that the BTB domain interacts with proteins involved in ubiquitination and protein chaperones (MYCBP2, HSPs) while the Kelch domain showed interactions with intermediate filaments, including NEFL and PRPH. These proteins may form a complex, as hypothesized in Fig 4E, consisting of a dimer of GIG, IFs interacting with the Kelch domain and MYCBP2 and/or HSPs binding to the Kelch domain for ubiquitin processing of IFs. MYCBP2 was the only E3 ligase to interact both with the BTB domain as well as with full-length GIG (at 99 and 94% CI, respectively). These findings certainly do not exclude Cul3 as an interactor, but open up the possibility that other E3 ligases may play a role in regulating intermediate filaments. The previous work showing proteasome inhibition reversed the clearance of IF proteins in GIG over expressing cells [15] which may suggest that small molecule proteasome activators like betulinic acid [23] might have the reverse effect, namely clearing IFs, although it is unclear what their specific protein target/s might be in the ubiquitination pathway.

In addition to possibly acting as an E3 ligase substrate adaptor, our finding that the BTB domain of GIG interacts with HSP90AA1/AB1 additionally raises the possibility that the fate of IFs bound to the Kelch domain may be controlled by HSPs. HSPs are known to act as critical regulators of protein quality control [24]. HSP90AA1 (inducible cytosolic HSP90) and HSP90AB1 (constitutive cytosolic HSP90) have been described to intersect with ubiquitination machinery and to tag misfolded proteins for degradation by the proteasome.

It is interesting to note that we did not identify the previously reported GIG binding partners microtubule-associated protein 1b light chain (MAP1b-LC), microtubule-associated protein 8 (MAP8), or tubulin-folding co-factor B (TBCB) [14, 25–27]. These proteins are present in HEK293 cells (S4 Fig), but their abundance or length of interaction with GIG may be below detection thresholds. Alternatively, the yeast two-hybrid screen used to identify these interactors may not identify the large complexes in which GIG participates.

Moreover, our MS screen did not detect an association between GIG and Cul3, a previously reported BTB binding partner [7, 28] suggesting that this interaction is either too transient or weak to be detected via MS. However, binding between overexpressed GIG and Cul3 was clearly apparent (S4 Fig) and VIM levels have been reported to increase upon treatment of GIG-overexpressing cells with the proteasomal inhibitor MG132 [15] suggesting that GIG degrades IFs at least in part by a proteasomal pathway. Our study refines and extends this model by identifying additional components of the protein degradation machinery recruited to BTB domains of GIG.

Our proteomic screen and the validation of the interactions with IFs were all done in HEK293 cells, which, although they express basal levels of some neuronal markers, are clearly distinct from the neurons affected in GAN patients. In addition, to identify Kelch interactors, it was necessary to use overexpression rather than the endogenous form of GIG for the initial screen. It will therefore be important in the future to determine whether motor/sensory neuron-specific interactors of GIG might explain the specificity of the observed clinical phenotype. Unfortunately, the extremely low levels of soluble IFs and GIG in human neurons make these experiments challenging.

In summary, our data provide a global compendium of proteins interacting with GIG. The unbiased proteomic analysis provides strong support to the model in which GIG serves as a linker between substrate proteins and protein degradation machinery. Critically, the study identified multiple intermediate filament proteins interacting with the substrate-binding Kelch domain, as well as several new potential partners of the BTB domain that might be involved in the degradation of GIG substrates. Deeper appreciation of the complexity of the GIG protein network may lead to identification of novel targets for the development of strategies for the clearance of accumulated IFs in GAN patients.

## Materials and Methods

### Protein isolation and Western Blot Analysis

Cells were isolated, suspended in RIPA lysis buffer supplemented with protease and phosphatase inhibitor cocktail (Roche, NY, USA), triturated and centrifuged at 10,000 g for 15 minutes at 4°C. 1–10 μg of total protein was separated on 10% SDS-polyacrylamide gels, transferred to a nitrocellulose membrane and probed with various primary antibodies, followed by horseradish-peroxidase-conjugated secondary antibody (1,:5000–10,000, for mouse or rabbit primaries respectively, Invitrogen), and visualized using ECL chemiluminescence (Pierce, IL, USA). Primary antibodies used in this study are GIG (1:150, a generous gift from Dr. Pascale Bomont), HISH3, (1:500, Millipore, MAB052), PRPH (1:5,000, kindly provided by Dr. Ron Liem).

### Affinity purification and mass spectrometry analysis

HEK293 cells were transfected with PGK::FLAG-GIG or PGK::FLAG-BTB constructs. 28 hours later, cells were lysed and immunoprecipitation was performed as previously described [29]. Targets were eluted by incubation with 3X flag peptide (Sigma). Samples were analyzed by Applied Biomics, Inc (Hayward, CA) via NanoLC-MS/MS after tryptic digestion, and only high confidence hits (CI > 95%) were considered for analysis. CRAPome analysis was performed as previously described [30]. DAVID analysis was performed using the published online tool [17]. For follow-up immunoprecipitation experiments, the same strategy was used to isolate flag-GIG. Endogenous GIG was immunoprecipitated using rabbit α GIG (NBP1-49924, Novus Biologicals). Endogenous VIM was immunoprecipitated using mouse α VIM (BD 550513 or AMF-17b). The mouse peripherin construct was a generous gift from Jean-Pierre Julienne (Université Laval).

### Pathway analysis

The Ingenuity Pathways Analysis (IPA) software was used to map the proteins interacting in experiments onto canonical pathways and functional networks. Lists of proteins for each of the conditions were uploaded for GIG (N = 79 proteins), BTB (N = 47), see main text for selection details. The pathway analysis included direct and indirect relationships. Tabulation and consolidation of results using Python and MySQL.

### qRT-PCR

Total RNA from HEK293 cells was isolated using Trizol LS (Invitrogen). 1 μg was treated with DNase (Invitrogen) and was subsequently used to synthesize cDNA with iScript (Bio-Rad). Quantitative RT-PCR was then performed using SYBR green (Bio-Rad) and the iCycler system (Bio-rad). Quantitative levels for all genes were normalized to endogenous GAPDH. Standard curves were run to ensure equal efficiency of all primers. Primer sequences are available upon request. Data were analyzed using the comparative quantitation by the 2^-ΔΔC^_T_ equation. ΔΔC_T_ = ΔC_T_ (sample)– ΔC_T_ (control), where ΔC_T_ is the threshold cycle (C_T_) value of the housekeeping gene GAPDH subtracted from the C_T_ value of the target gene.

## Supporting Information

S1 Fig Symbols from IPA networks. Symbol legend from IPA networks in shown in Fig 3 and S2 and S3 Figs.
(TIF)

S2 Fig Remaining top Kelch interaction networks. Displayed are the third (A), fourth (B) and fifth (C) ranked Kelch interactions networks.
(TIF)

S3 Fig Remaining top BTB interaction networks. Displayed are the third (A) and fourth (B) ranked BTB interactions networks.
(TIF)

S4 Fig Previously identified interactors of GIG. (A) HEK293 cell expression of MAP1B, MAP8 and TBCB as demonstrated by qPCR. (B) HEK293 cells were transfected with HA-Cul3 and GIG, and HA immunoprecipitation was performed, resulting in pulldown of GIG.
(TIF)

S1 Table Full list of hits from GIG proteomic screen. The raw data is included as well as subsequent filtering steps in sequential tabs. Data was parsed according to 98 and 80% confidence intervals and tabulated using Python and MySQL to identify Kelch and BTB-interacting proteins.
(XLSX)

S2 Table Complete DAVID ontology profile of Kelch hits. The final data set from S1 Table was processed using the DAVID functional annotation tool. Gene groups and enrichment scores are indicated and summarized in Fig 2D.
(XLSX)

## Abbreviations

ALS: amyotrophic lateral sclerosis
BACK: BTB/Kelch associated
BTB: Bric-a-brac, Tramtrack and Broad
CI: confidence interval
CNS: central nervous system
GAN: giant axonal neuropath
GIG: gigaxonin
HSP: heat shock protein
HSPA9: Heat shock 70 kDa protein 9
HSPD1: Heat shock 60 kDa protein 1
Hsp70: Heat shock 70 kDa protein
Hsp90: Heat shock 90 kDa protein
HSP90AA1/AB1: heat shock protein 90 alpha/beta
IFs: intermediate filaments
INA: α-internexin
IPA: Ingenuity Pathway Analysis
MALDI-TOF/TOF: matrix-assisted laser desorption/ionization-time of flight
MAP1b-LC: microtubule-associated protein 1b light chain
MAP8: microtubule-associated protein 8
MS: mass spectrometry
MYCBP2: MYC binding protein 2 and E3 ubiquitin protein ligase
Nano-LC: nano-liquid chromatography
NC: negative control
NEFH: neurofilament heavy chain
NEFL: neurofilament light chain
NEFM: neurofilament medium chain
PNS: peripheral nervous system
PRPH: peripherin
Rbx1: ring box protein 1
TBCB: tubulin-folding co-factor B
UBC: ubiquitin C
VIM: vimentin
