# Supplementary figures and images for: Kelch Domain of Gigaxonin Interacts with Intermediate Filament Proteins Affected in Giant Axonal Neuropathy
Source: PLoS One. 2015 Oct 13;10(10):e0140157. doi: 10.1371/journal.pone.0140157 (PMC4604155; doi:10.1371/journal.pone.0140157)

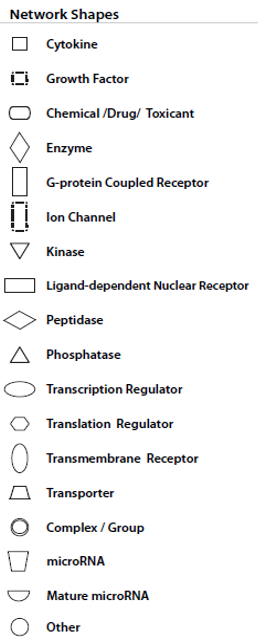

Supplement: S1 Fig — Symbol legend from IPA networks in shown in Fig 3 and S2 and S3 Figs. (TIF) [file pone.0140157.s001.tif]

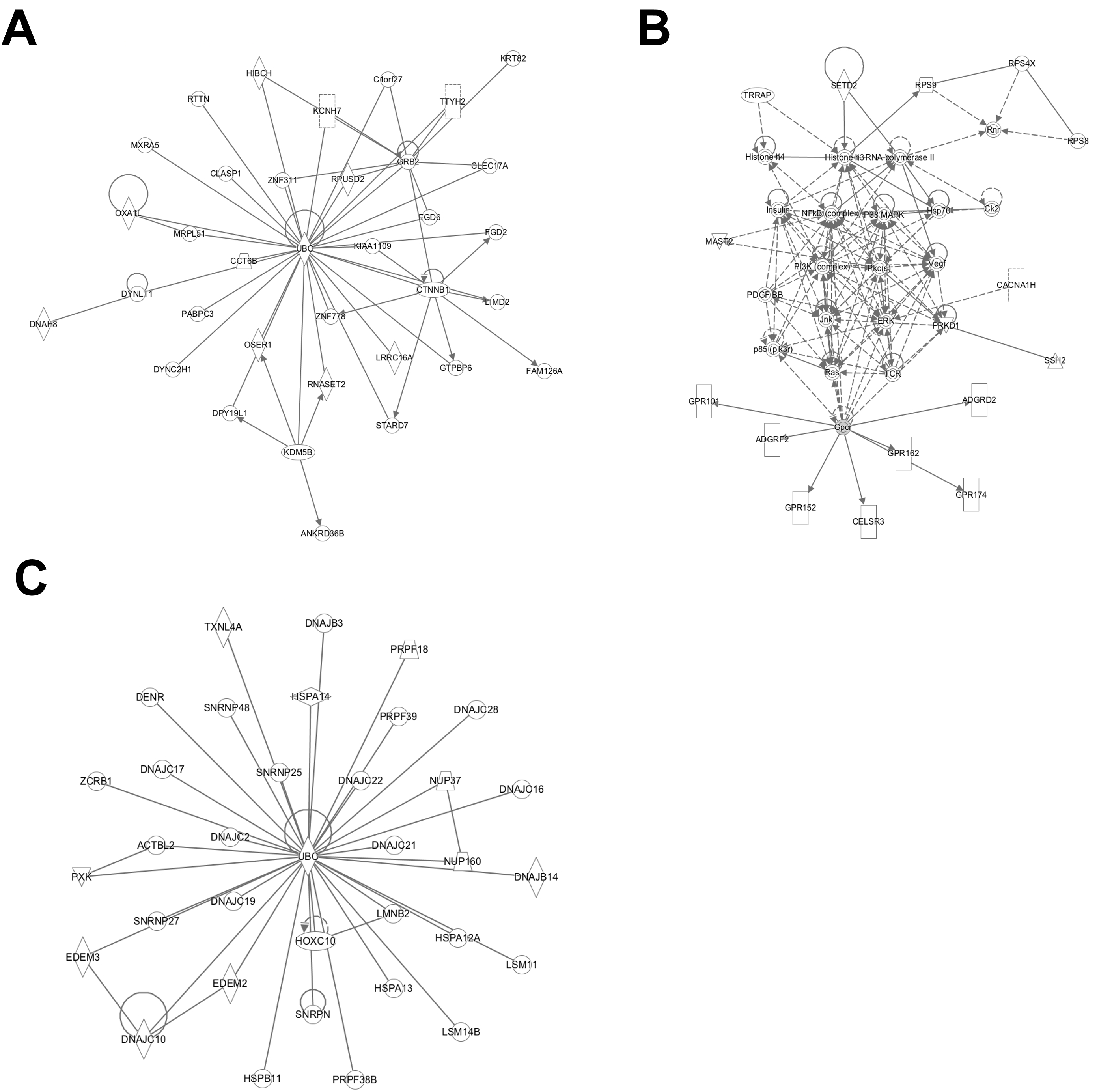

Supplement: S2 Fig — Displayed are the third (A), fourth (B) and fifth (C) ranked Kelch interactions networks. (TIF) [file pone.0140157.s002.tif]

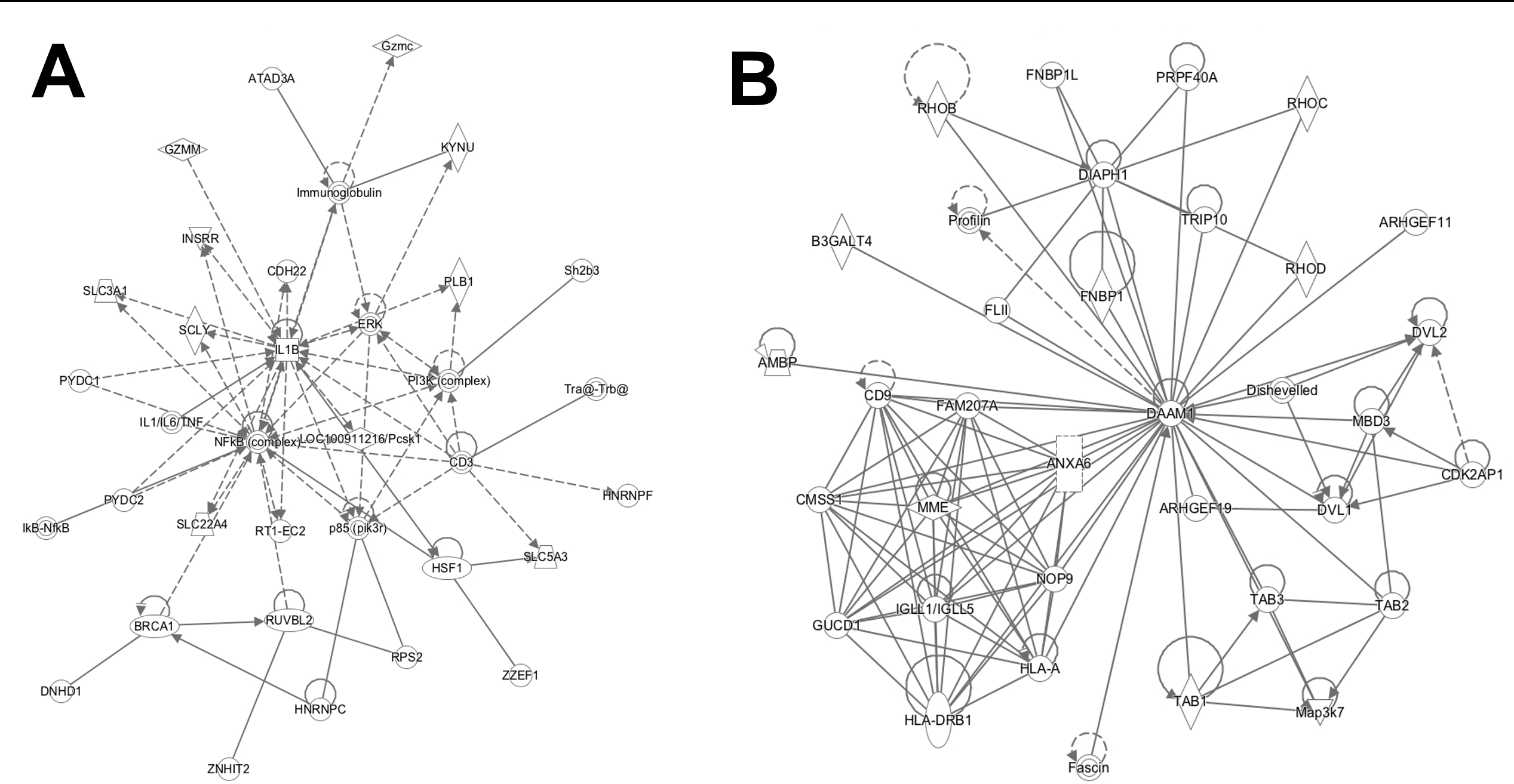

Supplement: S3 Fig — Displayed are the third (A) and fourth (B) ranked BTB interactions networks. (TIF) [file pone.0140157.s003.tif]

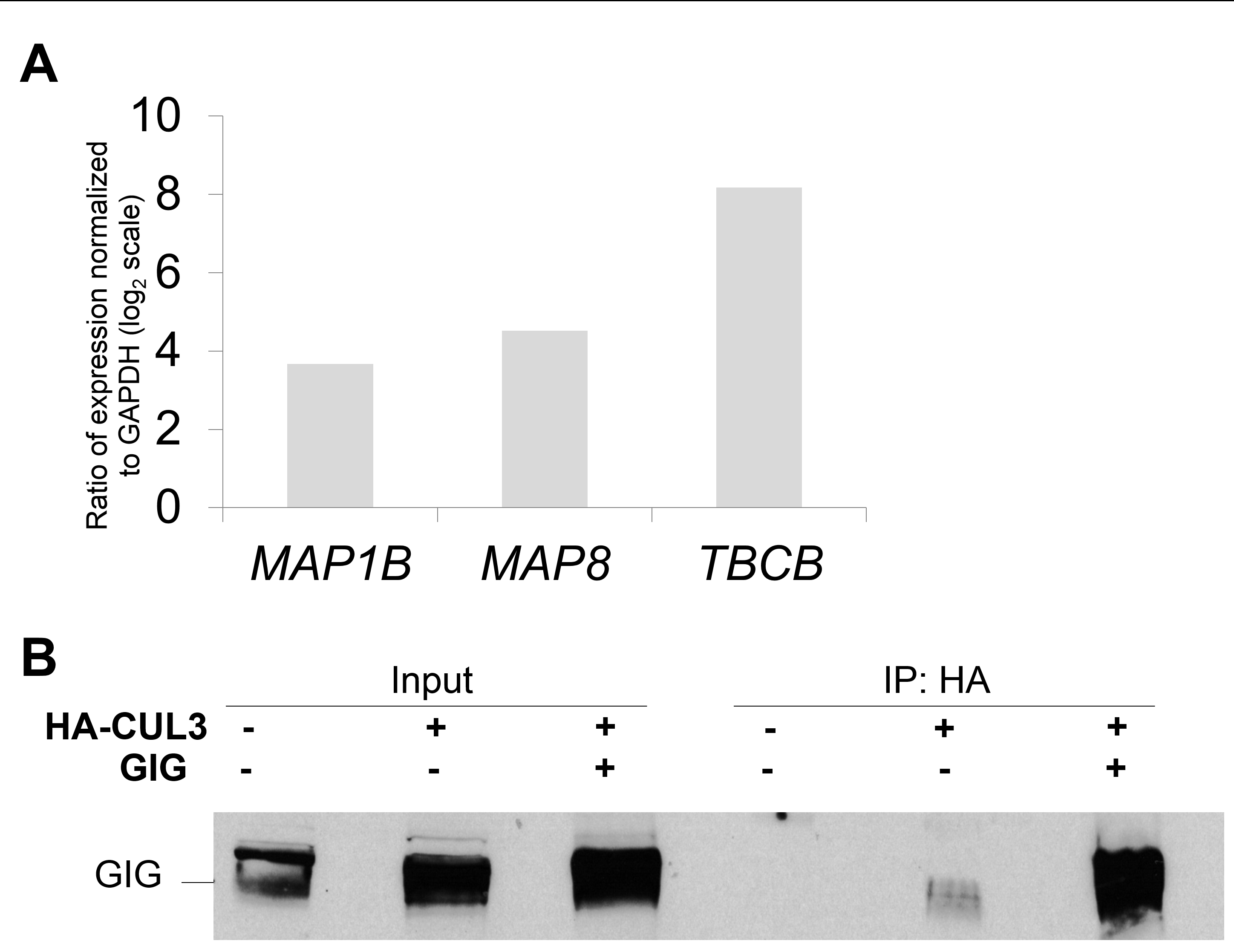

Supplement: S4 Fig — (A) HEK293 cell expression of MAP1B, MAP8 and TBCB as demonstrated by qPCR. (B) HEK293 cells were transfected with HA-Cul3 and GIG, and HA immunoprecipitation was performed, resulting in pulldown of GIG. (TIF) [file pone.0140157.s004.tif]
